# Visual Agnosia for Line Drawings and Silhouettes without Apparent Impairment of Real-Object Recognition: A Case Report

**DOI:** 10.3233/BEN-2009-0244

**Published:** 2009-12-07

**Authors:** Kotaro Hiraoka, Kyoko Suzuki, Kazumi Hirayama, Etsuro Mori

**Affiliations:** ^1^Department of Behavioral Neurology and Cognitive NeuroscienceTohoku University Graduate School of MedicineAoba-kuSendaiJapan; ^2^Department of Clinical NeuroscienceYamagata University Graduate School of MedicineYamagataJapan

**Keywords:** Visual agnosia, real objects, photographs, line drawings, silhouettes, cerebral infarction, posterior cerebral artery

## Abstract

We report on a patient with visual agnosia for line drawings and silhouette pictures following cerebral infarction in the region of the right posterior cerebral artery. The patient retained the ability to recognize real objects and their photographs, and could precisely copy line drawings of objects that she could not name. This case report highlights the importance of clinicians and researchers paying special attention to avoid overlooking agnosia in such cases. The factors that lead to problems in the identification of stimuli other than real objects in agnosic cases are discussed.

## 1. Introduction

Visual agnosia is a relatively rare clinical syndrome characterized by impairment in the visual recognition of objects. Typically, agnosic patients have no underlying neurosensorial deficits, and do not suffer from global cognitive impairment. They are easily able to identify by touch or literal definition objects that they cannot recognize visually.

Here, we report on a patient with visual agnosia for line drawings and silhouettes following an ischaemic stroke in the region of the right posterior cerebral artery. The patient retained the ability to recognize real objects and their photographs, and could precisely copy line drawings of objects that she could not name.

## 2. Case report

A 74-year-old, right-handed woman with 12 years of education was admitted to hospital having experienced topographical disorientation for three days. Her medical history included more than 30 years of treatment for hypertension, and cataract operations in both eyes.

When she got up one morning, she could not recognize where she was, a situation that resolved within a few hours. Later that day, she was unable to return to her flat from the nearby bus stop via the shortest route. On admission to hospital, neurological examinations revealed no abnormalities except bilateral superior altitudinal hemianopia, which was confirmed by Goldman perimetery. The patient's only problem on the ward was topographical disorientation, i.e. an inability to remember the locations of places she visited every day, such as the lavatory and rehabilitation room. She could identify faces and objects on the ward without difficulty.

Diffusion-weighted magnetic resonance images on admission showed an infarction in the region of the

ISSN 0953-4180/09/$17.00 © 2009 – IOS Press and the authors. All rights reserved

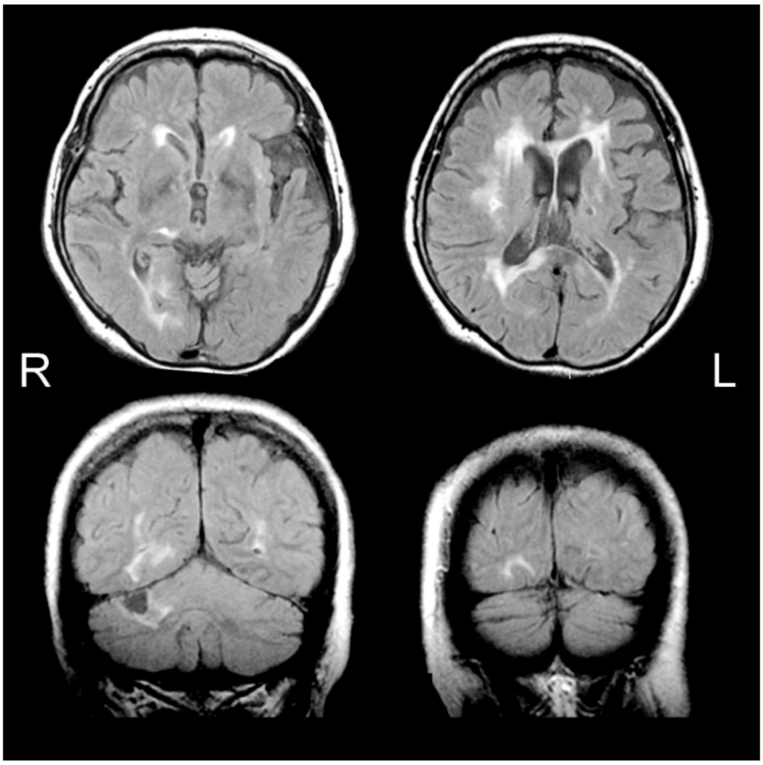

Fig. 1. FLAIR images of MRI. The lesion involved the subcortical white matter beneath the right fusiform and lingual gyri, and the right half of the splenium of the corpus callosum extending forward to the pulvinar. Old ischaemic lesions were also revealed in the white matter of the bilateral frontal lobes and right cerebellar hemisphere, and a subcortical lesion beneath the left lingual gyrus.

right posterior cerebral artery. The lesions involved the right occipital lobe and the right half of the splenium of the corpus callosum extending forward to the pulvinar. In addition to these acute lesions including the subcortical white matter of the right fusiform and lingual gyri, fluid-attenuated inversion recovery images revealed old ischaemic lesions affecting the white matter of the bilateral frontal lobes and the right cerebellar hemisphere, and a subcortical lesion beneath the left lingual gyrus (Fig. 1). Single photon emission computed tomography showed decreased accumulation in the medial part of the right occipital lobe, an inferior part of the right temporal lobe, the right cerebellar hemisphere, and the ventromedial part of the left occipital lobe.

Table 1 shows the results of standard neuropsychological examinations. The patient performed within the normal range in the Wechsler Adult Intelligent Scale-Revised [11,17], Western Aphasia Battery [6,13] and verbal fluency tests [1]. However, her performance on the Wechsler Memory Scale-Revised [14,18] and Rey Complex Figure Test [7] was poor, indicating amnesia predominantly for visual materials.

Table 1
Summary of neuropsychological test results

| Test | Result |
|---|---|
| Wechsler Adult Intelligent Scale-Revised | |
| Verbal IQ | 96 |
| Performance IQ | 70 |
| Full-scale IQ | 82 |
| Wechsler Memory Scale-Revised | |
| Verbal Memory Index | 75 |
| Visual Memory Index | 66 |
| Delayed Recall Index | 60 |
| Rey Complex Figure Test | |
| Copy | 32/36 |
| Immediate Recall | 0/36 |
| Delayed Recall | 1/36 |
| Recognition | 15/24 |
| Western Aphasia Battery | |
| Aphasia Quotient | 94.2 |
| Cortical Quotient | 94.1 |
| Verbal Fluency (Phoneme) | 12 (/1 min) |
| Verbal Fluency (Category) | 12 (/1 min) |

## 3. Experimental investigations

### 3.1. *Basic visual function*

Corrected visual acuity of the right and left eyes was 20/20 and 20/25, respectively. Although contrast sensi-

Table 2
Number (and percentage) of correct answers given by the patient and by age-matched control subjects in the visual recognition tasks

| Stimuli | Patient | Control subjects ($n = 5$) | |
|---|---|---|---|
| | | mean | SD |
| Real objects ($n = 20$) | 20 (100%) | 20 (100%) | 0.0 |
| Colour photographs of objects ($n = 12$) | 12 (100%) | 12 (100%) | 0.0 |
| Monochrome photographs of objects ($n = 12$) | 12 (100%) | 11.8 (98.3%) | 0.0 |
| Line drawings of objects ($n = 100$) | 81 (81.0%) | 99.6 (99.6%) | 0.6 |
| Silhouette pictures of objects ($n = 50$) | 41 (82.0%) | 48.8 (97.6%) | 1.2 |

SD: standard deviation.

tivity measured by a previously described method [10] was mildly impaired throughout the range of space-frequency, this impairment was not severe enough to disturb the patient's visual recognition of objects and drawings. In the City University Colour Vision Test [4], 3 out of 10 trials were incorrect, which suggested a tritan defect. The patient also made 3 errors in 13 trials of Ohkuma pseudoisochromatic plates [8], suggesting a deficiency in distinguishing red from green.

### 3.2. *Visual recognition*

The patient's visual recognition ability was examined using real objects, photographs, line drawings and silhouette pictures. The real objects consisted of 20 items in the object-naming task of the Western Aphasia Battery [6] such as a pencil, a key and a knife, which were placed one at a time on a table in front of the patient. The stimuli for the photographs were colour and monochrome pictures of 12 familiar objects such as a sunflower, a butterfly, an apple and an airplane. The stimuli for line drawings were 100 line drawings of familiar objects, which we previously developed for the picture-naming task [15]. All drawings were in high-contrast black against white background and contained some texture. The stimuli for the silhouette pictures were 50 common objects selected from the Snodgrass and Vanderwart norms [12], which were painted over with black. The categories of objects depicted in line drawings and silhouettes included fruit, clothing, animals, vehicles etc. The objects depicted in the photographs, line drawings and silhouettes were shown from usual viewpoints and were 12 cm high or wide at their maximum point. The patient was asked to name or describe these objects.

Table 2 shows the results of the visual recognition tasks carried out by the patient and by 5 healthy controls matched for age and education. The patient could provide the correct names of all objects shown as real objects or depicted in colour or monochrome photographs. In contrast, she had difficulty recognizing the line drawings of objects, and was unable to identify and name correctly 19% and 18% of objects depicted in line drawings and silhouettes, respectively. The errors in the test of line drawings and silhouettes, respectively, were classified as semantic (53%, 67%) when they had a semantic relationship with the stimulus; no response (26%, 0%) when the patient did not give any response; and visual (21%, 33%) when they had a visual but not a semantic similarity with the stimulus.

The patient could precisely copy line drawings of objects that she was unable to name. However, even after successful copying, she was unable to recognize the drawn objects (Fig. 2). With verbal descriptions of the objects, she could correctly name all 19 objects that she failed to recognize visually.

For the assessment of auditory recognition, the patient was asked to name the objects by listening to a specific sound, such as the sound of a moving train or the barking of dogs. She could recognize all 12 stimuli correctly. For the assessment of tactile recognition of objects, with her eyes closed, the patient touched objects with her hands and then named or described them, and successfully recognized all 10 stimuli.

## 4. Discussion

The patient showed topographical disorientation at onset, and visual agnosia, achromatopsia and memory disorder on detailed neuropsychological examination. An ischaemic lesion of the subcortical white matter of the right fusiform and lingual gyri, coexisting with an old subcortical lesion beneath the left lingual gyrus, resulted in bilateral superior altitudinal hemianopia, achromatopsia and visual agnosia.

The patient had difficulty recognizing line drawings and silhouettes. Following investigation of various aspects of basic visual functions, mild impairment of contrast sensitivity was detected, but her ability to copy pictures flawlessly indicated that this did not interfere with picture recognition. Mild achromatopsia was al-

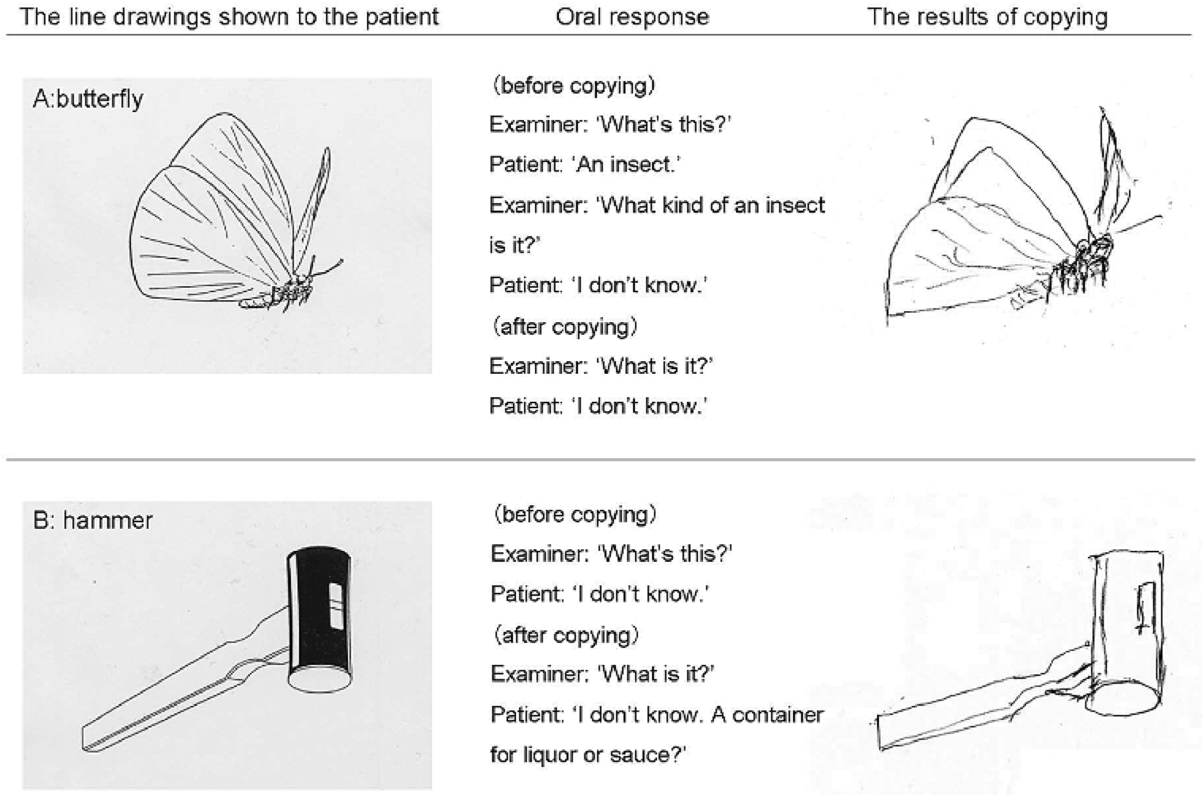

Fig. 2. The results of copying line drawings and oral responses before and after copying.

so detected. However, it did not impair the patient's ability to recognize line drawings and silhouettes, as these were monochrome. In view of the fact that she was able to recognize colour and monochrome photographs, which are visually much more complex than line drawings and silhouettes, it is clear that her performance was not being affected by the complexity of the pictures. These findings suggest that the patient's impaired recognition of line drawings and silhouettes was not due to a disorder of low-level visual functions. With verbal descriptions of objects, she could correctly name all the objects that she failed to recognize visually, which indicated that access to semantics from verbal input is spared. Based on these results and on the fact that tactile recognition and auditory recognition were preserved, the impaired recognition of line drawings and silhouettes must have been due to visual agnosia.

In contrast to agnosia for line drawings and silhouettes, impairment of real-object recognition was not apparent in either the patient's daily life or in our experimental investigations. In many reported cases of visual agnosia, there is impaired visual recognition of objects, which causes problems in daily life [2,9,16]. Although there is no convincing evidence that our patient's agnosia was restricted to line drawings and silhouettes, as the stimuli of the different tasks (i.e. recognition of real objects, colour and monochrome photographs, line drawings and silhouettes) were not the same in terms of numbers, familiarity, category etc., visual agnosia for real objects in this case was so subtle, if present at all, that it could not be observed in daily life and on routine psychological testing. Special attention should be paid so as not to overlook agnosia in similar cases.

For many patients with visual agnosia, recognition of real objects is superior to recognition of line drawings [3,5,9,16]. Some previous case studies of visual agnosia have shed light on the mechanism of the discrepancy between the recognition of real objects and the recognition of the same objects shown in two-dimensional formats. In a study by Humphrey et al. [5], an agnosic patient was shown real objects under different conditions and the same objects depicted in colour and monochrome photographs and in line drawings. The real objects were presented under four different viewing conditions: (i) full viewing, (ii) monocular viewing, in which one eye was covered, (iii) monochromatic viewing, in which the object was viewed through a red-coloured filter, and (iv) monocular viewing combined with monochromatic viewing. The accuracy of naming varied markedly over the different conditions of presentation, ranging from 71% correct in full viewing to only 11% correct in the case of line drawings. There was a regular decrease in the number of objects recognized by the patient as more surface information was removed. The study's authors suggested that the presence of surface properties, such as colour and texture, and depth information aided recognition.

In a single case study by Chainay et al. [2], an agnosic patient was shown objects and asked to name them in different conditions, i.e. coloured and monochrome photographs, monochrome line drawings, and correctly and incongruently coloured line drawings. Correct naming was worse with monochrome line drawings (30% correct) relative to all other conditions, even including incongruently coloured drawings (43–47% correct). From this result, the study's authors suggested that for their patient, who was achromatopsic, the colours, whether appropriate for the objects or not, provided segmentation cues about the surfaces and parts of the objects, since common surfaces and parts tend to have similar colour and luminance values. Another experiment was conducted in the same study to evaluate the contribution of three-dimensional cues to recognition. Real objects were presented within arm's reach (to enable binocular disparities) or at a greater distance (to reduce the effectiveness of binocular cues). In addition, the patient viewed the objects with his head free or in a fixed position (in the latter case, to remove motion parallax cues from head movements). Recognition performance did not differ significantly in conditions in which the objects were shown nearby with the patient's head free or fixed and when they were shown at a distance with the patient's head free (73–80% correct). Conversely, recognition performance was poorer when the objects were shown at a distance with the patient's head fixed (49% correct). These results suggest that depth information, obtained by both binocular disparity cues and head movements, facilitated object recognition. Overall, the authors concluded that information about depth and surface shading can contribute to object recognition when edge-based object recognition is impaired.

In the light of these studies, the discrepancy between the recognition of real objects and the recognition of the same objects shown in 2D formats in many cases of visual agnosia may be due to the difference in the number of visual cues for recognition in each format of the stimuli. Compared with real objects, photographs lack information about size and 3D shape that is gained from binocular disparity. Line drawings only have contour, and insufficient cues of internal details, texture and 3D shape. Compared to line drawings, silhouettes gain luminance, but lose cues of internal details, texture and 3D shape. In the condition in which the ventral visual pathway that plays a key role in object recognition is disordered, the paucity of visual cues may contribute significantly to the failure of recognition.

## Acknowledgment

This work was supported by Grant-in-Aid for Scientific Research on Priority Areas-System study on higher-order brain functions-from the Ministry of Education, Culture, Sports, Science and Technology of Japan (20020004).
